# The chemo- enzymatic synthesis of labeled l-amino acids and some of their derivatives

**DOI:** 10.1007/s10967-018-5932-z

**Published:** 2018-05-30

**Authors:** Małgorzata Pająk, Katarzyna Pałka, Elżbieta Winnicka, Marianna Kańska

**Affiliations:** 1 Department of Chemistry, Warsaw University, Pasteur 1 Str., 02-093 Warsaw, Poland; 2 Department of Biochemistry, 2nd Faculty of Medicine, Medical University of Warsaw, 61 Zwirki i Wigury Av., 02-091 Warsaw, Poland

**Keywords:** Amino acid, Bioamine, Deuterium, Enzyme, Labeling, Tritium

## Abstract

This review compiles the combined chemical and enzymatic synthesis of aromatic l-amino acids (l-phenylalanine, l-tyrosine, l-DOPA, l-tryptophan, and their derivatives and precursors) specifically labeled with carbon and hydrogen isotopes, which were elaborated in our research group by the past 20 years. These compounds could be then employed to characterize the mechanisms of enzymatic reactions via kinetic and solvent isotope effects methods.

## Introduction

This review deals with combined chemical and enzymatic synthesis of aromatic l-amino acids and bioamines labeled specifically with carbon and hydrogen isotopes. These compounds play an essential role in biochemical processes of life. Therefore, in the past the majority of very laborious syntheses have been carried out to provide these biologically active compounds, which were used as analytical, diagnostic, or therapeutic agents. However, the main impact on searches for new improved methods of synthesis comes from nuclear medicine, biochemistry, and pharmacy. Information on these methods are scattered, although a large knowledge may be taken starting from the large monograph published quite a long time ago [1], or from subsequently issued book [2–4]. In response to the growing demands for the labeled compounds, recently enzymatic methods were introduced, leading to the formation of needed biologically active products. However, there are no literature reviews devoted only to the synthesis of labeled compounds of particular relevance to the field of life science.

Our research group investigates the mechanisms of reactions catalyzed by enzymes. We employ isotopic techniques, particularly kinetic isotope effect (KIE) and solvent isotope effect (SIE) methods [5, 6], which require the use of selectively labeled compounds. For the abovementioned purposes the combined chemical and enzymatic synthesis of isotopomers of l-aromatic amino acids, its precursors, and derived bioamines, selectively labeled with isotopes of carbon and hydrogen were elaborated. In this paper we review previously published methods of synthesis of isotopomers of l-phenylalanine, l-tyrosine, l-DOPA, l-tryptophan, their derivatives, and precursors, all of which are specifically labeled with isotopes of hydrogen and carbon.

## Synthesis

### Synthesis of l-phenylalanine labeled with hydrogen and carbon isotopes

**Fig. 1:**
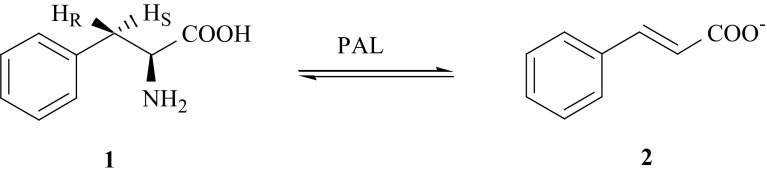
Reaction catalyzed by ammonia lyase

The synthesis of isotopomers of l-phenylalanine, l-Phe (**1**), specifically labeled with isotopes of carbon and hydrogen were elaborated by us to study the mechanism of elimination of ammonia from l-Phe catalyzed by enzyme phenylalanine ammonia lyase (PAL, EC 4.3.1.5), leading to formation of (*E*)-cinnamic acid (**2**) [7–12] according to Fig. 1.

**Fig. 2:**
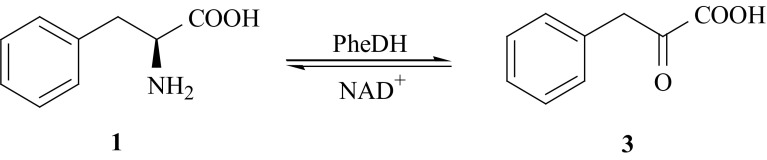
Oxidative deamination catalyzed by enzyme l-phenylalanine dehydrogenase

The metabolism of l-Phe is also connected with one of the human genetic disease—phenylketonuria (PKU), which is accompanied by elevated levels of l-Phe (**1**) metabolites such as phenylpyruvate and phenyllactate in body fluids. The knowledge about the mechanism of enzymatic conversion of l-Phe (**1**) into phenylpyruvic acid, PPA (**3**) is essential for proper therapy of PKU patients. One of the metabolic paths of conversion of (**1**) into (**3**) is reversible, oxidative deamination catalyzed by enzyme l-phenylalanine dehydrogenase (PheDH, EC 1.4.1.20) [13, 14] (Fig. 2).

The above two multistep reactions involve several intermediates, and therefore it is important to determine the structure of active complexes formed in the rate determining step. The number of arising questions can be minimized by determining kinetic isotope effects, KIE, of carbon 14, deuterium and tritium, as well as, the deuterium solvent isotope effects, SIE. Aforementioned studies require the use of the optically active forms of (**1**) specifically labeled with deuterium or tritium in desired (*3R*) and (*3S*) positions. The introduction of label in these specific positions only by chemical methods is a very tedious, time consuming, and sometimes even impossible, therefore, the combined chemical and enzymatic approaches were used.

For the preparation of labeled enantiomers of phenylalanine, the experimental procedures described in the literature resulted in multilabeled products or those labeled specifically with deuterium in irrelevant positions [15–19]. Also in the earlier reported studies on the synthesis of stereoisomers of [3-^2^H]- and [3-^3^H]-Phe the desired products were obtained as a result of tedious, multi step chemical synthesis [20–24]. Furthermore, often the enzymatic approach was applied to separate l- and d-isomers as the last step.

**Fig. 3:**
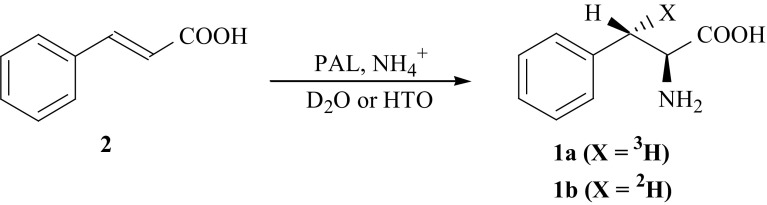
Synthesis of l-Phe isotopomers labeled with hydrogen isotopes in the (3*S*) position

For synthesis of specifically labeled isotopomer, [(*3S*)-^3^H]-l-Phe (**1a**) properties of the enzyme PAL were used. This enzyme, under proper conditions, catalyzes addition of ammonia to (*E*)-cinnamic acid (**2**) resulting in formation of l-Phe (**1**) [7]. The synthesis of (**1a**) was performed according to Fig. 3. Addition of ammonia to cinnamic acid, catalyzed by PAL, was carried out in the buffer containing tritiated water, HTO, leading to formation of (**1a**) [25, 26].

The same approach was taken to obtain deuterium labeled [(*3S*)-^2^H]-l-Phe (**1b**). In this case, addition of ammonia was carried out in fully deuterated phosphate buffer.

**Fig. 4:**
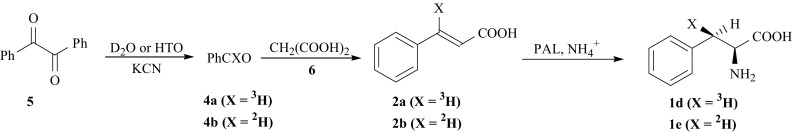
Synthesis of l-Phe isotopomers labeled with hydrogen isotopes in the (3*R*) position

The synthesis of tritiated isotopomer [(*3R*)-^3^H]-l-Phe (**1d)**, was carried out according to Fig. 4. The compound (**1d**) was obtained by combining chemical and enzymatic methods. In the first step, benzaldehyde (**4**) labeled with tritium in the formyl group {[7-^3^H]-benzaldehyde} (**4a**) was prepared. There are several synthetic protocols for obtaining the benzaldehyde, however, most of them require expensive reagents and long reaction time. The method chosen by us involved the step in which benzil, (PhCO)_2_ (**5**), was cleaved by cyanide ion in tritiated water, HTO. The tritiated benzaldehyde (**4a**) was condensed with malonic acid (**6**) in pyridine solvent using catalytic amounts of piperidine giving [3-^3^H]-cinnamic acid (**2a**). Compound **2a** then was converted into (**1d**) by addition of ammonia catalyzed by PAL [25] (Fig. 4).

Deuterium labeled [(*3R*)-^2^H]-l-Phe (**1e**) was synthesized the same way by cleaving benzil in heavy water (99.9% D_2_O) and adding ammonia to resulted [3-^2^H]-cinnamic acid (**2b**).

In the literature there are several procedures describing the synthesis of l-Phe labeled with isotopes of carbon. Isotopomers [3-^11^C]-dl-Phe [27] and [3-^14^C]-D-Phe [28] were obtained using multi step chemical path. Also, [3-^11^C]-l-Phe was synthesized via combined chemo- and enzymatic method [29]. ^13^C- or doubly labeled [^2^H, ^13^C]- phenylalanines were obtained using purified enzymes [24] or intact microorganisms [19, 30] in the key step of reaction. However, these methods yielded irreverently labeled products only, useful for spectroscopic studies.

**Fig. 5:**
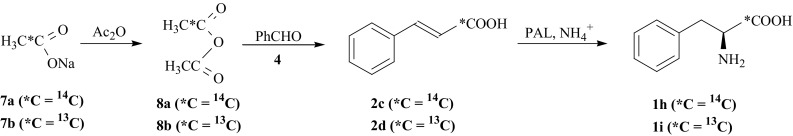
Synthesis of l-Phe isotopomers labeled with carbon isotopes

Our studies have required different isotopomers of l-Phe specifically labeled with isotopes of carbon in desired positions. For KIE studies the novel enzymatic pathways to obtain the desired labeled compounds i.e. [1-^14^C]-l-Phe (**1** **h**) and [1-^13^C]-l-Phe (**1i**) using sodium [1-^14^C]-acetate (**7a**) or sodium [1-^13^C]-acetate (**7b**) as a source of carbon label were elaborated [31]. The syntheses were carried out according to Fig. 5. Labeled sodium acetates (**7a** or 7**b**) were converted into labeled acetic anhydrides (**8a**, **8b**) and then reacted with benzaldehyde (**4**) yielding labeled [1-^14^C]- (**2c**) or [1-^13^C]-cinnamic acid (**2d**). These compounds were converted into desired isotopomers (**1h**) and (**1i**) by enzymatic addition of ammonia catalyzed by the enzyme PAL.

**Fig. 6:**
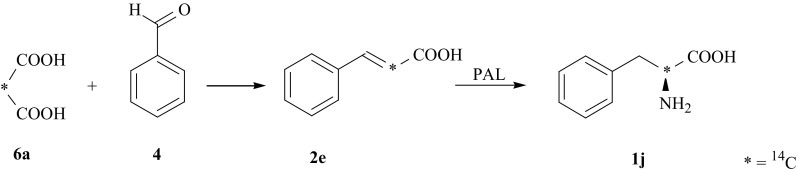
Chemo-enzymatic synthesis of [2-^14^C]-l-Phe

Another synthetic route to obtain the isotopomer of (**1**) specifically labeled with ^14^C, i.e., [2-^14^C]-l-Phe (**1j**), consists of combination of chemical and enzymatic methods [32]. As a source of ^14^C commercially available [2-^14^C]-malonic acid (**6a**) was used. Then the labeled [2-^14^C]-cinnamic acid (**2e**) was prepared by Knovenagel condensation. In the last step of the synthesis leading to the pure l-enantiomer of phenylalanine (**1j**), the activity of enzyme PAL was used (Fig. 6).

**Fig. 7:**
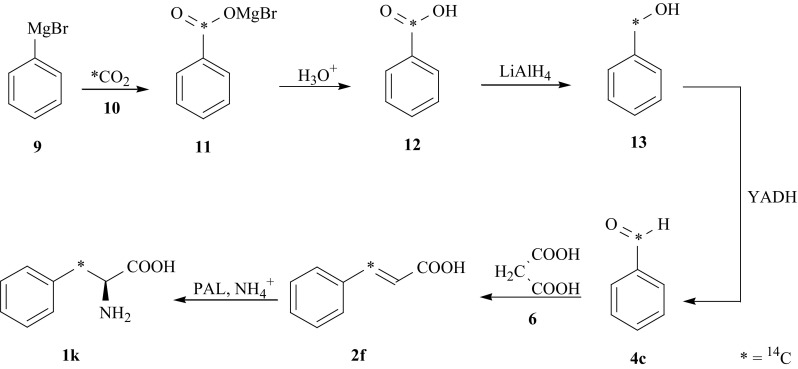
Chemo-enzymatic synthesis of [3-^14^C]-l-Phe

For synthesis of [3-^14^C]-l-Phe (**1k**), [1-^14^C]-benzaldehyde (**4c**) and unlabeled malonic acid (**6**) were applied. [1-^14^C]-Benzaldehyde (**4c**) was prepared using the following reaction sequence: carbonation of Grignard’s reagent PhMgBr (**9**) with [^14^C]carbon dioxide (**10**), hydrolysis resulted Ph^14^COOMgBr (**11**) to [7-^14^C]-benzoic acid (**12**), which was reduced with LiAlH_4_ yielded [7-^14^C]-benzyl alcohol (**13**). Next (**13**) was enzymatically converted into [1-^14^C]-benzaldehyde (**4c**) using yeast alcohol dehydrogenase (YADH, EC 1.1.1.1), which condensed with malonic acid (**6**) gave [3-^14^C]-cinnamic acid (**2f**). Finally, the addition of ammonia to (**2f**) catalyzed by PAL leads to (**1k**) (Fig. 7) [32, 33].

The characteristic of isotopomers of l-Phe (**1**) are collected in Table 1.

**Table 1:** The characteristic of l-Phe isotopomers

Compound	Specific activity (Bq/mmol)	Chemical yield (%)	References
[(*3S*)-^3^H]-l-Phe (**1a**)	9.3 × 10^8^	37.7	[25]
[(*3R*)-^3^H]-l-Phe (**1d**)	1.13 × 10^7^	34.3	[25]
[1-^14^C]-l-Phe (**1h**)	–	45.2	[31]
[1-^13^C]-l-Phe (**1i**)	–	48.8	[31]
[2-^14^C]-l-Phe (**1j**)	1.82 × 10^6^	46	[32]
[3-^14^C]-l-Phe (**1k**)	0.46 × 10^6^	46	[32]

### Synthesis of l-tyrosine labeled with hydrogen and carbon isotopes

**Fig. 8:**
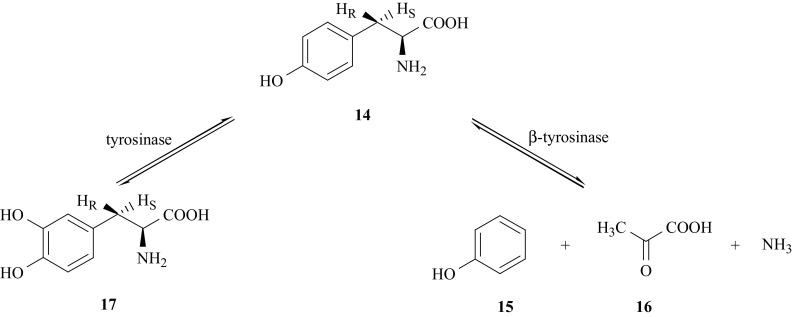
Reactions catalyzed by tyrosinase and β-tyrosinase

The metabolism of l-tyrosine, l-Tyr (**14**), is a key step in many biological processes of living organisms. A number of questions cannot be answered without understanding the mechanisms of the reversible conversion of l-Tyr (**14**) to phenol (**15**), pyruvate (**16**) and ammonia, a reaction that is catalyzed by the enzyme β-tyrosinase (tyrosine phenol lyase, EC 4.1.99.2) [34–38] and enzymatic conversion of l-Tyr (**14**) to l-DOPA (**17**), catalyzed by tyrosinase (EC 1.14.18.1) [39–41] (Fig. 8). Such studies using KIE and SIE methods [42–45] require different isotopomers of l-Tyr (**14**), specifically labeled with carbon and hydrogen isotopes in the positions of interest.

l-Tyrosine multilabeled with deuterium in several positions has been prepared by the different routes including chemical [46–48] and enzymatic [17, 49] mainly for spectroscopic studies. However, to study the mechanisms of enzymatic reactions, doubly labeled [3-^2^H/^3^H]-l-Tyr [50] and deuterated [(*2S*)-^2^H]-, [(*2R*)-^2^H]-l-Tyr [51] were obtained as a result of multistep chemical synthesis.

**Fig. 9:**
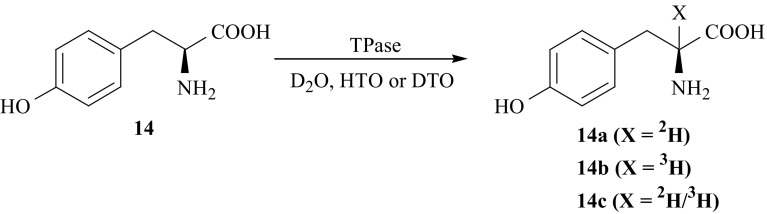
Enzymatic synthesis of l-Tyr isotopomers labeled with hydrogen isotopes in the second position of the side chain

For our KIE studies the isotopomers of l-Tyr (**14**), labeled in the second position of the side chain, i.e., [2-^2^H]-l-Tyr (**14a**) and [2-^3^H]-l-Tyr (**14b**) were synthesized using the simpler way (Fig. 9). The label (deuterium or tritium) was introduced into 2-position of (**14**) by enzymatic isotopic exchange between incubation medium (containing D_2_O or HTO) and (**14**), catalyzed by enzyme tryptophanase (TPase, EC 4.1.99.1) from *E*. *coli* [52]. Under some conditions this enzyme causes labilization of hydrogen attached to α-carbon of many native l-amino acids and facilitates the H/D(T) exchange [53]. Doubly labeled isotopomer [2-^2^H/^3^H]-l-Tyr (**14c**) was obtained the same manner using fully deuterated buffer with DTO added.

**Fig. 10:**
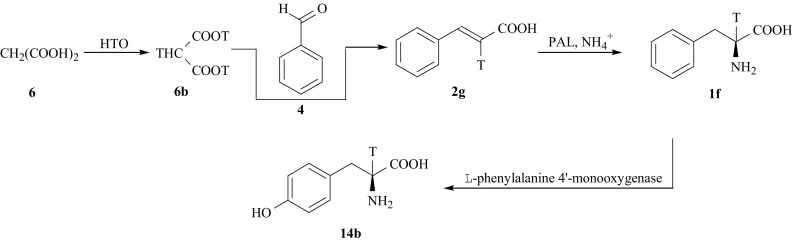
Chemo-enzymaic synthesis of [2-^3^H]-l-Tyr

[2-^3^H]-l-Tyr (**14b**) was also synthesized from [2-^3^H]-l-Phe (**1f**), according to the reaction route shown in Fig. 10. The tritium label was introduced into the methylene group of malonic acid (**6**), as a result of isotopic exchange of (**6**) and tritiated water at elevated temperature [54]. Knovenagel condensation of [2-^3^H]-malonic acid (**6b**) with benzaldehyde (**4**) leads to [2-^3^H]-cinnamic acid (**2g**), which in turn was converted by enzyme PAL to [2-^3^H]-l-Phe (**1f**). Finally, tritiated l-Phe incubated in medium containing the enzyme l-phenylalanine 4′-monooxygenase (EC 1.14.16.1) from rat liver, produces [2-^3^H]-l-Tyr (**14b**). To stimulate the hydroxylation of l-Phe to l-Tyr, the reaction was carried out in the presence of d,l-6-methyl-5,6,7,8-tetrahydropterine (cofactor) and d,l-dithiothreitol. The medium also contained the enzyme catalase (EC 1.11.1.6) that protects lTyr from H_2_O_2_ formed during the course of incubation. The general protocol of this step was described earlier [55].

**Fig. 11:**
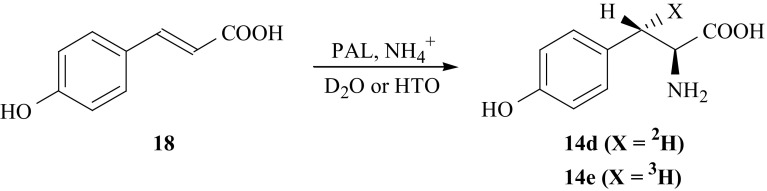
Synthesis of l-Tyr isotopomers labeled with hydrogen isotopes in the (3*S*) position

The enzyme PAL was also used for the synthesis of [(*3S*)-^2^H]- (**14d**) and [(*3S*)-^3^H]-lTyr (**14e**) [52, 55] (Fig. 11). Under proper conditions this enzyme catalyzes addition of ammonia and hydrogen isotope (deuterium or tritium, depending on incubation medium) into *pro*-*S* position of *p*-coumaric acid (**18**) yielding (**14d**) or (**14e**). The yield of this synthetic route is very small, however it is the simplest way to obtain [(*3S*)-^2^H]- (**14d**) and [(*3S*)-^3^H]-lTyr (**14e**).

**Fig. 12:**
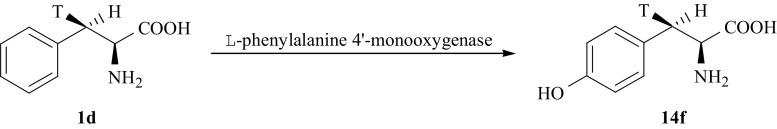
Synthesis of l-Tyr isotopomers labeled with hydrogen isotopes in the (3*R*) position

[(*3R*)-^3^H]-lTyr (**14f**) was synthesized by enzymatic hydroxylation of [(*3R*)-^3^H]-lphenylalanine (**1d**) obtained earlier [25] catalyzed by l-phenylalanine 4′-monooxygenase (Fig. 12). The hydroxylation of l-Phe to l-Tyr was carried out in the presence of d,l-6-methyl-5,6,7,8-tetrahydropterine (cofactor) and the enzyme catalase (EC 1.11.1.6) [55].

**Fig. 13:**
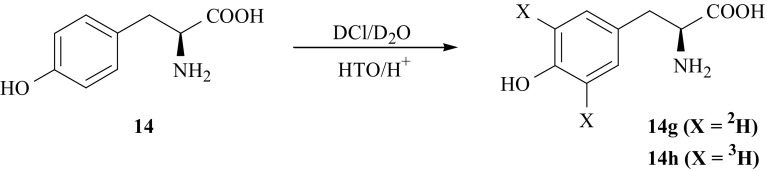
Synthesis of l-Tyr isotopomers labeled with hydrogen isotope in the 3′ and 5′ positions of aromatic ring

Isotopomers of l-tyrosine (**14**) labeled with deuterium in the 3′ and 5′ positions of the ring were obtained using isotopic exchange between heavy water and l-tyrosine. Under the acid catalyzed conditions, at elevated temperature, the exchange between D_2_O and l-Tyr (**14**) introduces deuterium exclusively into *orto* position [56, 57] (respectively to ring hydroxyl group) in (**14**), yielding [3′,5′-^2^H_2_]-l-Tyr (**14g**) [58, 59]. The same method was used to synthesize [3′,5′-^3^H_2_]-l-Tyr (**14h**). As a source of hydrogen isotope, tritiated water was used (Fig. 13).

**Fig. 14:**
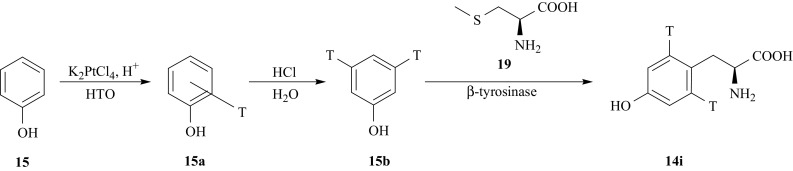
Synthesis of [2′,6′-^3^H_2_]-l-Tyr

Synthetic route of [2′,6′-^3^H_2_]-l-Tyr (**14i**) which consists of a combination of chemical and enzymatic methods [60] is shown in Fig. 14. First, the key intermediate i.e., [3,5-^3^H_2_]-phenol (**15b**) was obtained as a result of H/T exchange between phenol (**15**) and tritiated water. The literature data [56, 57, 61] show that phenol can be catalytically exchanged with deuterated or tritiated water selectively in the *o*- and *p*-positions or *per* labeled. By the reverse acid catalyzed exchange of uniformly tritiated phenol [U-^3^H]-PhOH (**15a**) with water it is possible to prepare [3,5-^3^H_2_]-phenol (**15b**), which in turn condensed with *S*-methyl-l-cysteine (**19**) using the enzyme *β*-tyrosinase (EC 4.1.99.2) from *Citrobacter freundii* yielded (**14i**).

In the literature there are reports of preparation of isotopomers of l-Tyr labeled with stable and radioactive carbon isotopes using classical chemical methods. Doubly labeled stereoisomers, i.e., *threo*- and *erythro*-[1-^13^C, 2,3-^2^H_2_]-l-Tyr, used for subsequent spectroscopic studies, were afforded in the multistep chemical synthesis [19]. Similarly, the pure chemical approach was applied for synthesis o [2-^11^C]-l-Tyr [62]. The demand for compounds labeled with short-lived ^11^C that are used as a diagnostic in nuclear medicine (i.e., positron emission tomography, PET) has prompted the efforts to synthesize amino acids labeled with this nuclide. Using ^11^CO_2_ as a source of label and applying the combined chemo- and multienzymatic methods the following isotopomers labeled in side chain were obtained: [1-^11^C]-l-Tyr [63], [2-^11^C]-l-Tyr [64] and [3-^11^C]-l-Tyr [43].

**Fig. 15:**
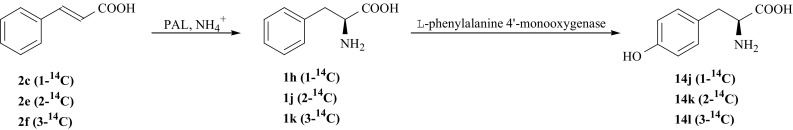
Synthesis of [1-^14^C]-, [2-^14^C]- and [3-^14^C]-l-Tyr

For our KIE studies three isotopomers of l-Tyr (**14**) specifically labeled with ^14^C in the 1-, 2- and 3-positions of the side chain {[1-^14^C]- (**14j**), [2-^14^C]- (**14k**) and [3-^14^C]-l-Tyr (**14l**)}, have been prepared using combined chemical and multienzymatic methods. For these syntheses, as intermediates, isotopomers of [1-^14^C]- (**2c**) [2-^14^C]- (**2e**), and [3-^14^C]-cinnamic acid (**2f**), have been converted into [1-^14^C]- (**1h**), [2-^14^C]- (**1j**), and [3-^14^C]-l-phenylalanine (**1k**), in the presence of the enzyme PAL (Fig. 15). In the next step, labeled l-Phe was oxidized to l-Tyr using an enzyme phenylalanine 4′-monooxygenase from rat liver [52, 65]. The hydroxylation of l-Phe to l-Tyr was carried out in the presence of a cofactor and the enzyme catalase (EC 1.11.1.6) that protects l-Tyr from hydrogen peroxide formed during incubation. The general route for the synthesis of labeled l-Tyr is shown in Fig. 15.

**Fig. 16:**
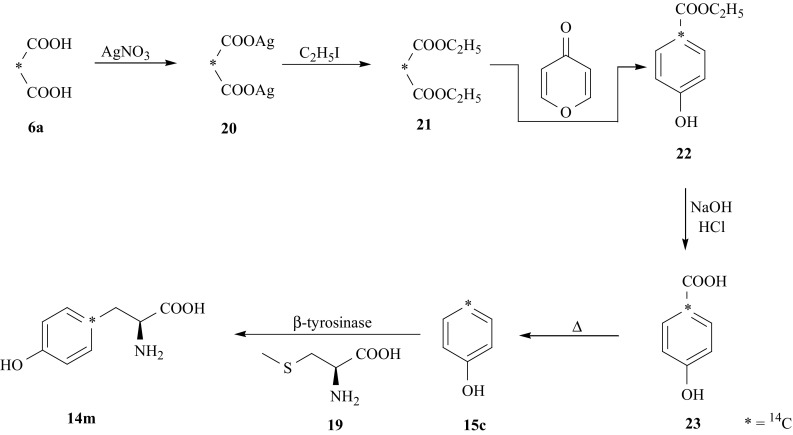
Synthesis of [1^′^-^14^C]-l-Tyr

Another compound, [1′-^14^C]-l-Tyr (**14m**), specifically labeled with ^14^C in the 1^′^ position in the ring, has been prepared in 6 step reaction sequence [66]. For this synthesis, as a starting substrate and a source of ^14^C label, [2-^14^C]-malonic acid (**6a**), was used. It was converted via its silver salt (**20**), in diethyl [2-^14^C]-malonate (**21**) [67]. The ring closure reaction of (**21)** with 4*H*-pyran-4-on afforded ethyl [1^′^-^14^C]-*p*-hydroxybenzoate (**22**), which was hydrolyzed to [1′-^14^C]-*p*-hydroxybenzoic acid (**23**). Its thermal decomposition yielded [4-^14^C]-phenol (**15c**) [68], which in turn was coupled [42] with *S*-methyl-l-cysteine (**19**) catalyzed by the enzyme β-tyrosinase from *Citrobacter freundii* yielding desired [1^′^-^14^C]-l-Tyr (**14m**) (Fig. 16).

The characteristic of l-Tyr (**14**) isotopomers are collected in Table 2.

**Table 2:** The characteristic of l-Tyr isotopomers

Compound	Specific activity (Bq/mmol)	Chemical yield (%)	References
[2-^3^H]-l-Tyr (**14b**)	3.58 × 10^8^	–	[52]
[(*3S*)-^2^H]-l-Tyr (**14d**)	–	1	[52]
[(*3S*)-^3^H]-l-Tyr (**14e**)	3.7 × 10^8^	–	[52]
[(*3R*)-^3^H]-l-Tyr (**14f**)	4.64 × 10^6^	–	[55]
[3′,5′-^3^H_2_]-l-Tyr (**14** **h**)	5.5 × 10^6^	75	[58]
[2′,6′-^3^H_2_]-l-Tyr (**14i**)	6.27 × 10^7^	18	[60]
[1-^14^C]-l-Tyr (**14j**)	6.8 × 10^6^	–	[65]
[2-^14^C]-l-Tyr (**14k**)	1.88 × 10^6^	–	[65]
[3-^14^C]-l-Tyr (**14l**)	2.8 × 10^6^	–	[52]
[1′-^14^C]-l-Tyr (**14m**)	1.83 × 10^6^	15	[66]

### Synthesis of l-DOPA labeled with hydrogen and carbon isotopes

**Fig. 17:**
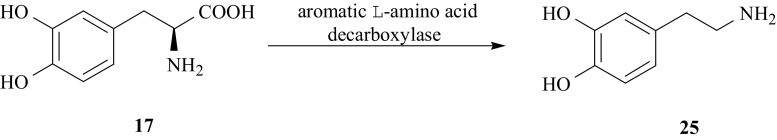
Enzymatic decarboxylation of l-DOPA

l-DOPA (3′,4′-dihydroxy-l-phenylalanine) (**17**), plays a significant role in many metabolic processes [69]. It is a precursor of biogenic amine—dopamine, DA, (**25**)—an important neurotransmitter in the nervous system of mammals. DA is formed in the brain as a result of decarboxylation of l-DOPA catalyzed by enzyme aromatic l-amino acid decarboxylase (EC 4.1.1.28) [70, 71] (Fig. 17). The mechanism of decarboxylation is not clear up to now, so for KIE and SIE studies specifically labeled isotopomers of l-DOPA are needed.

The original literature data concerning the synthesis of dl-DOPA specifically labeled with deuterium and tritium in different positions of ring and side chain are dated [72, 73] and yielded products useless for biological studies.

**Fig. 18:**
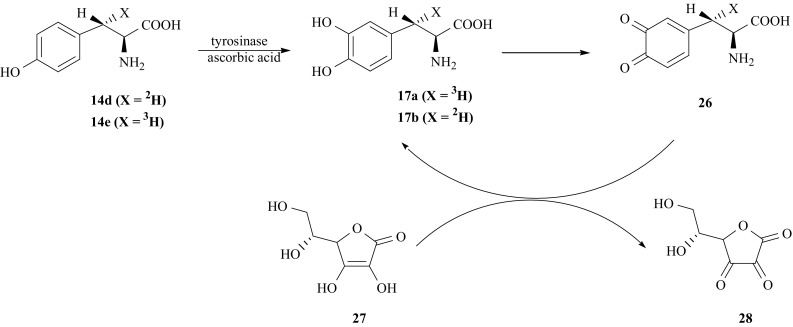
Synthesis of [(3*S*)-^2^H]- or [(3*S*)-^3^H]-l-DOPA

Needed for our purpose isotopomer [(*3S*)-^3^H]-l-DOPA (**17a**), selectively labeled with tritium in the (*3S*) position of the side chain, was obtained from the appropriate isotopomer of l-Tyr (**14e**) via enzymatic pathway [58] (Fig. 18). Enzyme tyrosinase from mushrooms *Neurospora Crassa* (EC 1.14.18.1) selectively introduces a hydroxyl group into the 3′-ring position of l-Tyr (**14**) [74], and also immediately mediates oxidation of l-DOPA to dopaquinone (**26**) [75]. However, in the presence of ascorbic acid (**27**), the oxidation of l-DOPA is a reversible process [76]. Ascorbic acid reduces dopaquinone to l-DOPA, and itself undergoes oxidation to dehydroascrobic acid (**28**).

For the synthesis of isotopomer [(*3S*)-^2^H]-l-DOPA (**17b**) labeled with deuterium in (*3S*) position of the side chain instead of (**14e**) as starting substrate (**14d**) was used (Fig. 18).

**Fig. 19:**
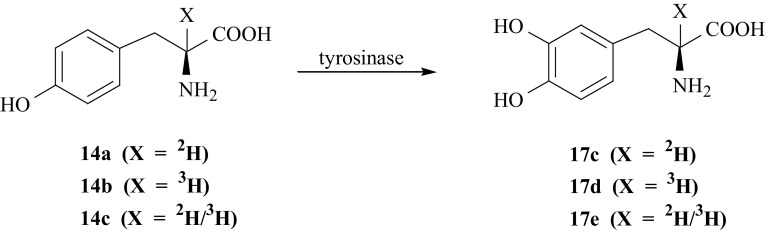
Enzymatic synthesis of isotopomers of l-DOPA labeled with hydrogen isotopes in the second position of the side chain

The same enzyme tyrosinase was used to obtain isotopomers of l-DOPA selectively labeled in the second position of the side chain, i.e., [2-^2^H]- (**17c**), [2-^3^H]- (**17d**) and [2-^2^H/^3^H]-l-DOPA (**17e**). Appropriate isotopomers of l-Tyr (**14a**, **14b**, **14c**) [52, 59, 77] were converted to l-DOPA by enzymatic hydroxylation catalyzed by enzyme tyrosinase in presence of ascorbic acid (Fig. 19).

**Fig. 20:**
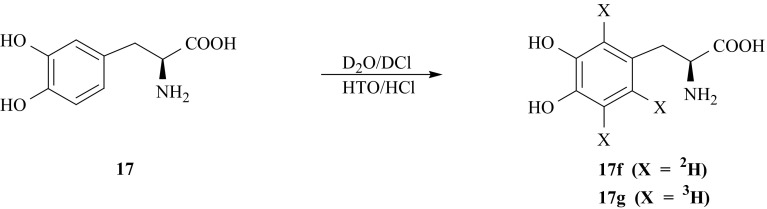
Synthesis of l-DOPA isotopomers labeled with hydrogen isotopes in the aromatic ring

The ring deuteration of l-DOPA (**17**) was carried out using acid catalyzed isotope exchange method at elevated temperature [78] (Fig. 20). No significant change of proton NMR signal integrations, corresponding to methylene and methine groups of the side chain, have been noticed in the course of experiments. The incorporation of deuterium takes place only into the aromatic ring of l-DOPA (**17**) yielding [2′,5′,6′-^2^H_3_]-l-DOPA (**17f**). Also the rates of H/D exchange are practically the same for the protons in 2′, 5′, and 6′ ring positions. Tritiation of (**17**) carried out in the same conditions using HTO as a source of ^3^H-label leads to [2′,5′,6′-^3^H_3_]-l-DOPA (**17** **g**).

l-DOPA labeled with ^14^C in carboxyl group, needed as internal radiometric standard, was synthesized [79] from [1-^14^C]-l-Tyr (**14j**) according to Fig. 18. The literature data concerning the chemical and combined chemo- enzymatic synthesis of l-DOPA bearing ^11^ C-label are very tedious and were designed to obtain the products for PET diagnosis. Chemical [80, 81] and chemo-enzymatic [63] routes are applied for synthesis of [1-^14^C]-l-DOPA, as well as for obtaining of [2-^11^C]-l-DOPA [64, 82]. The old paper reports on the synthesis of uniformly ring labeled [U-^14^C]-l-DOPA using [U-^14^C]-phenol as a substrate [83].

The characteristic of l-DOPA (**17**) isotopomers are collected in Table 3.

**Table 3:** The characteristic of l-DOPA isotopomers

Compound	Specific activity (Bq/mmol)	Chemical yield (%)	References
[(*3S*)-^3^H]-l-DOPA (**17a**)	3 × 10^8^	–	[58]
[2-^2^H]-l-DOPA (**17c**)	–	31	[77]
[2-^3^H]-l-DOPA (**17d**).	7.73 × 10^7^	46	[77]
[2-^2^H/^3^H]-l-DOPA (**17e**).	3.6 × 10^6^	43	[77]
[2′,5′,6′-^3^H_3_]-l-DOPA (**17** **g**)	1.57 × 10^8^	91	[78]

### Synthesis of l-tryptophan labeled with hydrogen and carbon isotopes

**Fig. 21:**
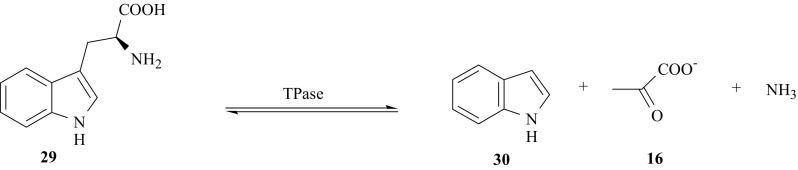
Enzymatic decomposition of l-Trp catalyzed by tryptophanase

The important metabolic reaction of l-tryptophan, l-Trp (**29**) in living organisms is its decomposition to the corresponding indole (**30**), pyruvate (**16**), and ammonia. This reaction is catalyzed by the enzyme tryptophanase (l-tryptophan indole lyase, TPase, EC 4.1.99.1) [84–86] (Fig. 21).

Under some experimental conditions the enzyme TPase catalyses the condensation of the indonyl moiety [(**30**) or 5-hydroxyindole (**31**)] with *S*-methyl-l-cysteine (**19**), leading to the synthesis of (**29**) or 5′-hydroksy-l-tyrptophan, 5′-OH-l-Trp (**32**), respectively [87]. The enzymatic labilization of hydrogen attached to the α-carbon (occurred in this reaction) facilitates the H/D or H/T exchange with the solvent (deuterated or tritiated water in this case).

The suggested mechanism of decomposition of l-Trp postulates proton transfer from the side chain to the C-3 carbon atom of the indole ring. This hypothesis should be verified by measuring the KIE for deuterium, tritium and carbon-14, as well as, the deuterium solvent isotope effects, SIE. For such kind of studies there is a need for isotopomers of l-Trp and 5′-OH-l-Trp specifically labeled with deuterium and tritium at the α-carbon position. Unfortunately, while the literature provides several synthetic methods leading to preparation of different isotopomers of tryptophan and its hydroxyl derivative labeled with deuterium and tritium specifically or nonspecifically, these reports are of little value for this purpose. Perdeuterated in indonyl moiety [^2^H_5_]-l-Trp [88] and [4,5,6,7 -^2^H_4_]-l-Trp were obtained by H/D exchange in D_2_O/CF_3_COOD solvent [46]. The [(*2RS*)-^2^H]-Trp was afforded by exchange with D_2_O by racemization/acylation procedure. This intermediate was resolved with acylase yielding [(*2S*)-^2^H]-Trp [89]. Four isotopomers of l-Trp labeled with deuterium specifically in indole ring have been obtained by coupling labeled indoles with l-serine catalyzed by extracts of *E. coli* cells containing enzyme tryptophan synthetase [90]. Also, the various isotopomers of tryptophan labeled with deuterium and tritium at the 2- and 3-positions of side chain were synthesized by chemical methods [91–93]. [5′-^2^H]-dl-Trp and [5′-^3^H]-dl-Trp were synthesized by reduction of 5-bromo-dl-Trp with gaseous deuterium or tritium [94]. In turn, 5′-hydroxy-[4′-^3^H]-dl-Trp was obtained by H/D exchange between 5′-hydroxy-dl-Trp and HTO [94]. 5′-Hydroxy-[4′-^3^H]-Trp was prepared by enzymatic hydroxylation of [4′-^3^H]-Trp [95]. In addition, the isotopomers doubly labeled with deuterium and ^13^C were prepared [19, 96] for spectroscopic studies.

**Fig. 22:**
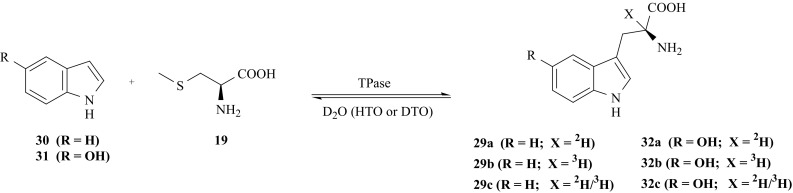
Synthesis of l-Trp and 5′-OH-l-Trp labeled with hydrogen isotopes at the α-carbon position

The coupling reaction (Fig. 22) was used by us to obtain l-Trp (**29**) and 5′-OH-l-Trp (**32**) labeled with the isotopes of hydrogen at the α-carbon position [97]. For the synthesis of [2-^2^H]-l-Trp (**29a**), and 5′-OH-[2-^2^H]-l-Trp (**32a**), all reagents were dissolved in fully deuterated phosphate buffer. For the synthesis of [2-^3^H]-l-Trp (**29b**), and 5′-OH-[2-^3^H]-l-Trp (**32b**) the reaction was carried out in phosphate buffer contained tritiated water (HTO). Doubly labeled [2-^2^H/^3^H]-l-Trp (**29c**), and 5′-OH-[2-^2^H/^3^H]-l-Trp (**32c**) have been obtained using a medium composed with fully deuterated phosphate buffer to which DTO was added. In all cases 2-mercaptoethanol was used to prevent the growth of bacteria and fungi during incubation.

**Fig. 23:**
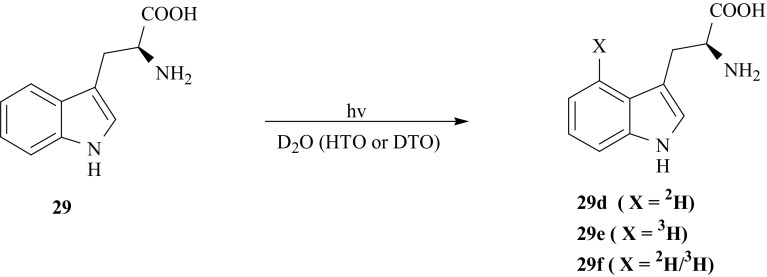
Synthesis of l-Trp labeled with hydrogen isotopes in the 4′ position of the aromatic ring

The isotopomer [4′-^2^H]-l-Trp (**29d**) was obtained by irradiation of the unbuffered solution of l-Trp (**29**) in heavy water with light from a 250 W mercury lamp filtered by Pyrex glass [98, 99]. The rate and degree of deuterium incorporation was monitored by ^1^H NMR spectroscopy. The results obtained in the course of deuteration allowed us to elaborate the exchange procedures for indole ring tritiation of (**29**). The tritiated [4′-^3^H]-l-Trp (**29e**) was obtained in one-step H/T exchange between (**29**) and tritiated water irradiated with UV light. The doubly labeled [4′-^2^H/^3^H-]-l-Trp (**29f**) was obtained in the same way by tritiation of deuterated (**29d**) dissolved in DTO (Fig. 23).

**Fig. 24:**
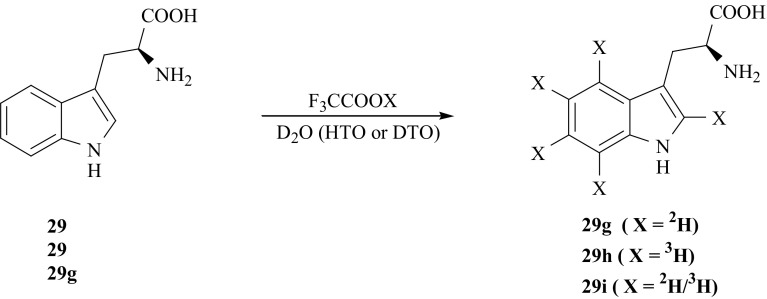
Synthesis of l-Trp uniformly labeled with hydrogen isotopes in the aromatic ring

The deuterated in the whole indole ring isotopomer, i.e., [2′,4′,5′,6′,7′-^2^H_5_]-l-Trp (**29g**) was obtained as a result of H/D exchange between (**29**) dissolved in a mixture of D_2_O and CF_3_COOD (1: 2, v/v). The reaction was carried out in darkness for 3 days at room temperature [47, 100]. The obtained product was isolated and the exchange procedure was repeated twice. After each step the extent of deuterium enrichment of (**29g**) was checked by means of ^1^H NMR. The whole indole ring tritiated [2′,4′,5′,6′,7′-^3^H_5_]-l-Trp (**29h**) was obtained as a result of one-step isotope exchange between (**29**) and the CF_3_COOH/HTO mixture. Also, the doubly labeled [2′,4′,5′,6′,7′-(^2^H/^3^H)_5_]-l-Trp **(29i**) was synthesized by one-step D/T exchange between deuterated (**29g**) and DTO [99] (Fig. 24).

For purposes of KIE studies, the specifically labeled with ^14^C isotopomers of l-Trp and 5′-OH-l-Trp were needed as an internal radiometric standard. In the literature there are descriptions of several methods of synthesis of the l-Trp and 5′-OH-l-Trp labeled with ^11^C or ^13^C. The ^13^C-indoles have been converted to the corresponding isotopomers of l-Trp using *E. coli* cells containing enzyme tryptophane synthetase [90, 101–103]. Also, the ^11^C-labeling of l-Trp [63, 104–106] have been reported for tumor diagnosis using PET. Additionally, several tedious chemical procedures of synthesis the different isotopomers of ^14^C-labeled dl-Trp have been described about 50 years ago [1].

**Fig. 25:**
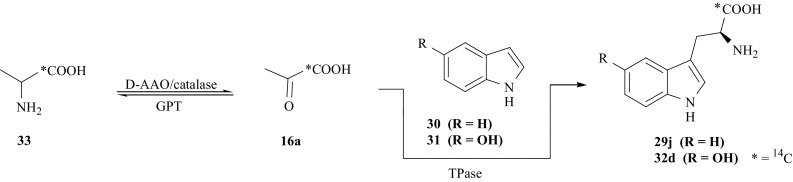
Synthesis of [1-^14^C]-l-Trp and 5′-OH-[1-^14^C]-l-Trp

[1-^14^C]-l-Trp (**29j**), and 5′-OH-[1-^14^C]-l-Trp (**32d**) specifically labeled with ^14^C in the carboxyl group, have been prepared using a combination of chemical and multienzymatic methods [107]. For this synthesis we applied, as an intermediate, a racemic mixture of [1-^14^C]-dl-alanine (**33**), which was obtained in a multistep synthesis that has been previously reported [108, 109]. ^14^CO_2_ (**10**) was used as a source of ^14^C-label. It has been converted in turn into (**33**) via [1-^14^C]-propionic acid (carbonation of the Grignard reagent C_2_H_5_MgI with ^14^CO_2_ and decomposition of the complex formed), 2-bromo[1-^14^C]propionic acid, followed by ammonolysis. In a one-pot multienzymatic synthesis (**33**) was converted into [1-^14^C]-pyruvic acid (**16a**) using the enzymes: D-amino acid oxidase (D-AAO, EC 1.4.3.3), catalase (EC 1.11.1.6) and glutamic-pyruvate transaminase (GPT, EC 2.6.1.2) [110]. In turn (**16a**) was coupled with indole (**30**) or 5-hydroxyindole (**31**) by the enzyme TPase (EC 4.1.99.1) giving (**29j**) or (**32d**), respectively (Fig. 25). Enzyme D-AAO rapidly transforms d-alanine to pyruvic acid only, therefore, to avoid the loss of half of radioactivity from the l-enantiomer we also used the second enzyme, GPT, converting l-alanine into pyruvic acid only. The medium also contained the enzyme catalase (EC 1.11.1.6) (removing the H_2_O_2_ formed) and coenzymes for D-AAO and GPT, i.e., flavin adenine dinucleotide, FAD, and pyridoxal 5′-phosphate, PLP, respectively [107].

**Fig. 26:**
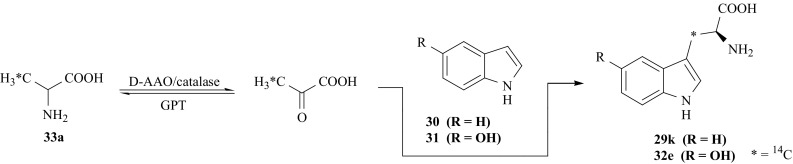
Synthesis of [3-^14^C]-l-Trp and 5′-OH-[3-^14^C]-l-Trp

In the same manner the isotopomers of [3-^14^C]-l-Trp (**29k**) and 5-OH-[3-^14^C]-l-Trp (**32e**) were obtained in one-pot multienzymatic synthesis in presence of the same four enzymes as above, and by using as substrates [3-^14^C]-dl-alanine (**33a**) and indole (**30**) or 5-hydroxyindole (**31**) respectively [111] (Fig. 26).

The characteristic of l-Trp (**29**) and 5′-OH-l-Trp (**32**) isotopomers are collected in Table 4.

**Table 4:** The characteristic of l-Trp and 5′-OH-l-Trp isotopomers

Compound	Specific activity (Bq/mmol)	References
of [2-^3^H]-l-Trp (**29b**)	4.5 × 10^6^	[97]
[2-^2^H/^3^H]-l-Trp (**29c**)	4.4 × 10^6^	[97]
[4′-^3^H]-l-Trp (**29e**)	8.9 × 10^7^	[99]
[4′-^2^H/^3^H-]-l-Trp (**29f**)	4.25 × 10^7^	[99]
[2′,4′,5′,6′,7′-^3^H_5_]-l-Trp (**29h**)	3 × 10^8^	[99]
[2′,4′,5′,6′,7′-(^2^H/^3^H)_5_]-l-Trp (**29i**)	1.4 × 10^8^	[99]
[1-^14^C]-l-Trp (**29j**)	1.03 × 10^6^	[107]
5′-OH-[2-^3^H]-l-Trp (**32b**)	4.38 × 10^6^	[97]
5′-OH-[2-^2^H/^3^H]-l-Trp (**32c**)	4.2 × 10^6^	[97]
5′-OH-[1-^14^C]-l-Trp (**32d**)	1.02 × 10^6^	[107]

### Synthesis of dopamine labeled with hydrogen isotopes

The biogenic amine, dopamine, DA, (**25**) plays an important role in many physiological functions as a neurotransmitter in the nervous system of mammals [112, 113]. DA (**25**) is also involved as a precursor in the synthetic enzymatic route of the other catecholamines as noradrenaline (**34**) and adrenaline (**35**) [114, 115].

**Fig. 27:**
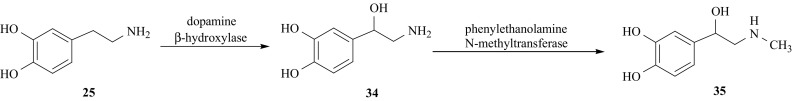
Enzymatic route of noradrenaline and adrenaline

The mechanism of β-hydroxylation of DA, leading to formation of noradrenaline, catalyzed by the enzyme dopamine β-hydroxylase (EC 1.14.17.1) (Fig. 27) are not completely clear up to now.

The literature data on the synthesis of labeled DA is very old and scarce. Dideutero [2-^2^H_2_]-DA was obtained by reduction of 3,4-dimethoxyphenylacetonitrile with LiAlD_4_ as [1-^2^H_2_]-DA was prepared from homoveratric acid by incorporation of deuterium into the side chain with exchange procedure [116]. The different isotopomers of DA tritiated in the 2- and 3-positions were obtained from (dihydroksyphenyl)ethyl alcohols as the result of three step chemical procedures [117]. Also, the very old data reports on chemo-enzymatic preparation of DA labeled with deuterium and tritium in the side chain [118, 119]. Deuterated [(*1S*)-^2^H]-DA and [(*1R*)-^2^H]-DA were obtained by enzymatic decarboxylation of [2-^2^H]-l-DOPA and l-DOPA, respectively [120].

Therefore, to study processes in Fig. 27 using KIE and SIE methods, a new simpler synthesis of deuterium or tritium labeled isotopomers of DA was elaborated.

**Fig. 28:**
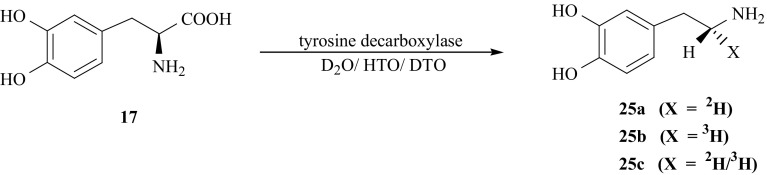
Enzymatic synthesis of dopamine isotopomers labeled with hydrogen isotopes in (1*R*) position

Isotopomers of (**25**) specifically labeled in the side chain, i.e., [(*1R*)-^2^H]- (**25a**) and [(*1R*)-^3^H]-DA (**25b**) were obtained by enzymatic decarboxylation of l-DOPA (**17**) catalyzed by the enzyme tyrosine decarboxylase (EC 4.1.1.25) from *Steptococcus faecalis* [121], and carried out in fully deuterated or tritiated medium respectively [122]. [(*1R*)-^2^H/^3^H]-DA (**25c**) was synthesized in fully deuterated incubation medium with DTO added [77] (Fig. 28).

**Fig. 29:**
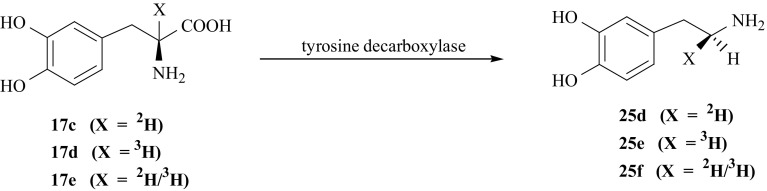
Enzymatic synthesis of dopamine isotopomers labeled with hydrogen isotopes in (1*S*) position

Previous studies have shown that enzymatic decarboxylation of l-amino acids occurs with retention of configuration at the α-carbon [123, 124]. This fact has been used to obtain two (*1S*)-isotopomers of (**25**) labeled with deuterium or tritium by enzymatic decarboxylation of specifically labeled isotopomers of l-DOPA (**17**) i.e., [2-^2^H]- (**17c**) and [2-^3^H]- (**17d**) and [2-^2^H/^3^H]-l-DOPA (**17e**) obtained earlier. According to this rule, the deuterium atom at C_α_ retains (*1S*)-configuration in [(*1S*)-^2^H]-DA (**25d**) obtained by enzymatic decarboxylation of (**17c**). Consequently when (**17d**) and (**17e**) are the substrates—[(*1S*)-^3^H]- (**25e**) and [(*1S*)-^2^H/^3^H]-DA (**25f**) were obtained [77] (Fig. 29). For these reactions enzyme tyrosine decarboxylase (EC 4.1.1.25) was used.

**Fig. 30:**
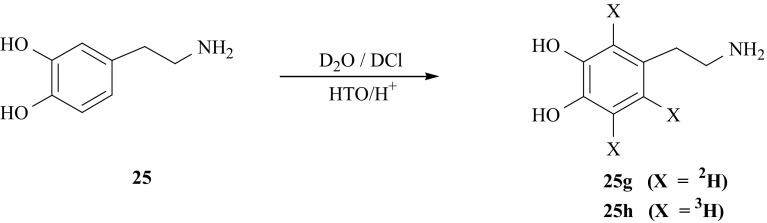
Synthesis of dopamine isotopomers labeled with hydrogen isotopes in aromatic ring using isotopic exchange

Isotopomers of DA (**25**) ring labeled with hydrogen isotopes, i.e., [2′,5′,6′-^2^H_3_]- (**25g**) and [2′,5′,6′-^3^H_3_]-DA (**25** **h**) were obtained using isotopic exchange method between heavy water and (**25**). Under the acid catalyzed conditions, at elevated temperature, the exchange between D_2_O and (**25**) takes place only into the aromatic ring [122] (Fig. 30). Tritiation of (**25**) was carried out in the same conditions but instead of D_2_O, tritiated water was used.

**Fig. 31:**
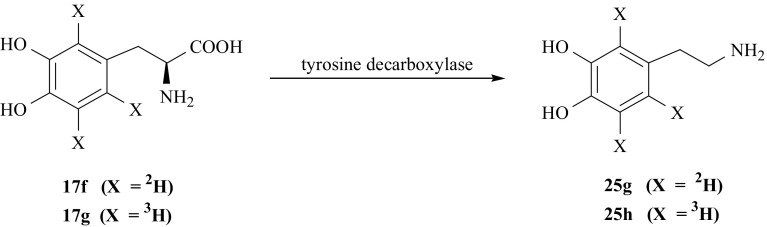
Enzymatic synthesis of dopamine isotopomers labeled with hydrogen isotopes in aromatic ring

For the synthesis of DA ring labeled with hydrogen isotopes, we have also used previously synthesized isotopomers of l-DOPA (**17**) [78]. [2′,5′,6′-^2^H_3_]- (**25g**) and [2′,5′,6′-^3^H_3_]-DA (**25h**) were obtained by enzymatic decarboxylation of [2′,5′,6′-^2^H_3_]- (**17f**) and [2′,5′,6′-^3^H_3_]-l-DOPA (**17g**), respectively, in presence of enzyme tyrosine decarboxylase (Fig. 31).

The characteristic of DA (**25**) isotopomers are collected in Table 5.

**Table 5:** The characteristic of DA isotopomers

Compound	Specific activity (Bq/mmol)	Chemical yield (%)	References
[(*1R*)-^2^H]-DA (**25a**)	–	81	[122]
[(*1R*)-^3^H]-DA (**25b**)	3.05 × 10^7^	82	[122]
[(*1R*)-^2^H/^3^H]-DA (**25c**)	2.33 × 10^7^	63	[77]
[(*1S*)-^2^H]-DA (**25d**)	–	80	[77]
[(*1S*)-^3^H]-DA (**25e**)	7.75 × 10^6^	78	[77]
[(*1S*)-^2^H/^3^H]-DA (**25f**)	3.5 × 10^6^	84	[77]
[2′,5′,6′-^2^H_3_]-DA (**25g**)	–	83.5	[78]
[2′,5′,6′-^3^H_3_]-DA (**25h**)	1.56 × 10^8^	63.5	[78]

### Synthesis of tyramine labeled with hydrogen isotopes

**Fig. 32:**
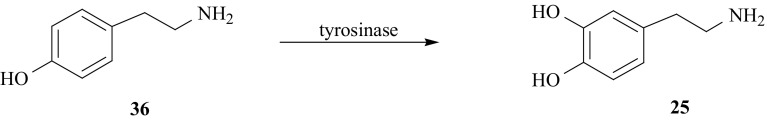
Synthesis of dopamine catalyzed by tyrosinase

Tyramine, TA (**36**), a biogenic amine, plays an important role in many metabolic processes. It is one of the trace amines in the central nervous system in humans [125, 126]. TA may also be a substrate for enzymatic hydroxylation to another important neurotransmitter such as DA (**25**), catalyzed by enzyme tyrosinase (EC 1.14.18.1), Fig. 32.

Some isotopomers of TA labeled with deuterium, tritium and ^14^C have been obtained during the study on the stereochemistry of enzymatic elimination of ammonia [127] and decarboxylation of l-Tyr [51]. Unfortunately, these chemical multistep syntheses are very labor intensive. For our purposes, to better understand the reaction of hydroxylation, specifically labeled isotopomers of (**36**), needed for KIE and SIE studies, were synthesized.

**Fig. 33:**
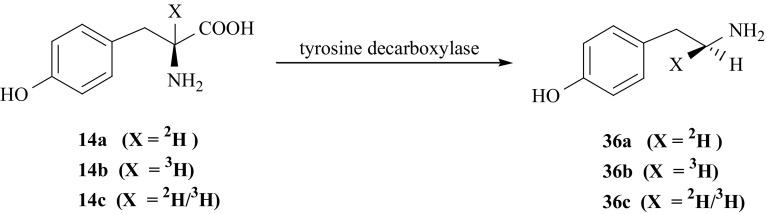
Enzymatic synthesis of tyramine isotopomers labeled with hydrogen isotopes in (1*S*) position

TA (**36**), specifically labeled with hydrogen isotopes in (*1S*) position, was obtained by enzymatic decarboxylation of labeled l-Tyr, catalyzed by tyrosine decarboxylase [59]. In the course of decarboxylation of l-Tyr (**14**) labeled in the 2-position of side chain, a solvent proton replaces the carboxyl group with retention of configuration [123, 124]. Therefore, the products obtained by enzymatic decarboxylation of isotopomers of l-Tyr {(**14a**), (**14b**), and (**14c**)} retain the label (deuterium or tritium) at configuration *S*, yielding [(*1S*)-^2^H]- (**36a**), [(*1S*)-^3^H]- (**36b**), and [(*1S*)-^2^H/^3^H]-TA (**36c**) (Fig. 33).

**Fig. 34:**
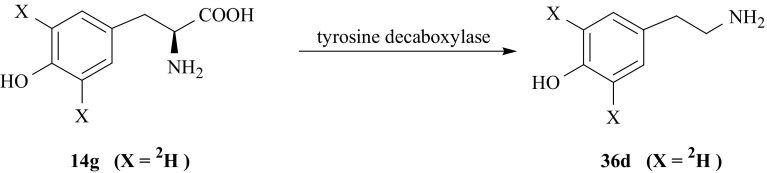
Enzymatic synthesis of tyramine isotopomer labeled with deuterium in 3′ and 5′ positions

TA ring labeled with hydrogen isotopes, i.e., [3′,5′-^2^H_2_]-TA (**36d**) was synthesized via two different routes. In the first, enzymatic decarboxylation of labeled l-Tyr (**14g**), catalyzed by tyrosine decarboxylase (EC 4.1.1.25), gives desired product (**36d**) [59] (Fig. 34).

**Fig. 35:**
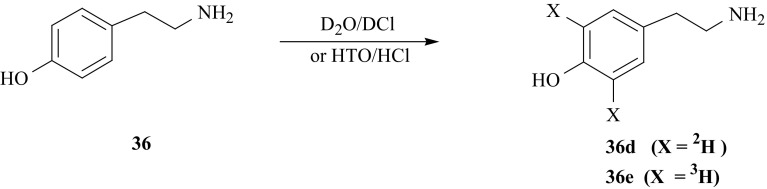
Synthesis of tyramine isotopomers labeled with hydrogen isotopes in aromatic ring using isotopic exchange

In the second direct synthetic route, deuterated [3′,5′-^2^H_2_]-TA (**36d**), as well as, tritiated [3′,5′-^3^H_2_]-TA (**36e**), were obtained in the course of acid catalyzed isotopic exchange carried out between deuterated or tritiated water and unlabeled tyramine (Fig. 35). In this case, the deuterium or tritium is incorporated exclusively into 3′ and 5′ ring position of TA (**36**).

The characteristic of TA (**36**) isotopomers are collected in Table 6.

**Table 6:** The characteristic of TA isotopomers

Compound	Specific activity (Bq/mmol)	Chemical yield (%)	References
[(*1S*)-^2^H]-TA (**36a**)	–	80	[59]
[(*1S*)-^3^H]-TA (**36b**)	3.66 × 10^7^	60	[59]
[(*1S*)-^2^H/^3^H]-TA (**36c**)	4.1 × 10^7^	66	[59]
[3′,5′-^2^H_2_]-TA (**36d**)	–	99	[59]
[3′,5′-^3^H_2_]-TA (**36e**)	6.9 × 10^7^	74	[59]

### Synthesis of histamine labeled with hydrogen isotopes

**Fig. 36:**
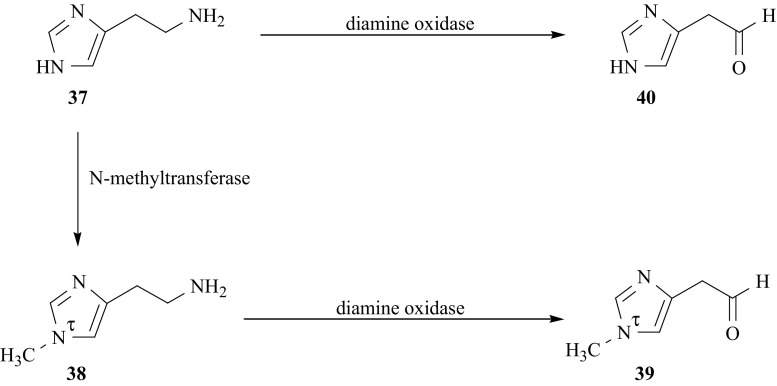
The fragment of metabolic pathway of histamine

The biogenic amine histamine, HA (**37**) plays an important role in various physiological function as a key mediator of cell growth, gastric secretion, acute allergic inflammation, and neurotransmitter for blood pressure [128–130]. In humans and experimental animals HA is mainly metabolized on the two pathways, Fig. 36 [131–133]. In humans about three quarters of HA is methylated to *N*^τ^-methylhistamine, τMeHA (**38**) by enzyme N-methyltransferase (EC 2.1.1.8), and subsequently this intermediate is oxidized to N^τ^-methylimidasole acetalaldehyde (**39**) by enzyme diamine oxidase (DAO, EC 1.4.3.6). The remaining quarter of HA, however, is indirectly biotransferred into imidasole acetalaldehyde (**40**) by DAO. (According to the recommendation of IUPAC [134], the nitrogen atoms of the imidasole ring are denoted by π and τ, carbon atoms in the side chain as α, and β and ring carbon atoms as 2, 4, 5).

Despite of many studies the mechanism of the removal of excess of HA (**37**) from human body is not completely understood. Therefore, we planned experiments to investigate some details of methylation and oxidation reactions presented in Fig. 36, by applying the KIE and SIE methods. For this kind of study the isotopomers of HA and N-methyl-HAs specifically labeled with deuterium and tritium are needed. In the literature there is description of the synthesis of τMeHA and πMeHA tritiated selectively in the methyl group [135]. The product obtained consists of two (τ and π) isomers, which separation was unsuccessful. Also, the preparation of tritiated (N^τ^-C[^3^H_3_])-HA from [^3^H]CH_3_I by chemical method is described [136], as well as the synthesis of deuterated (N^τ^-C[^2^H_3_])-HA [137].

**Fig. 37:**
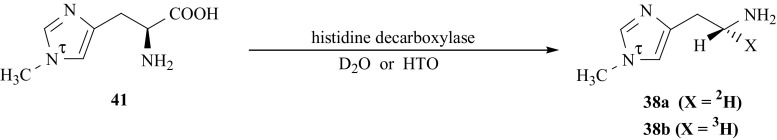
Enzymatic synthesis of τMeHA labeled with hydrogen isotopes in (*αR*) position

For KIE assays isotopomers [(*αR*)-^2^H]- (**38a**) and [(*αR*)-^3^H]-τMeHA (**38b**) specifically labeled with deuterium and tritium were obtained by enzymatic decarboxylation of *N*^τ^-methyl-l-histidine (**41**), catalyzed by the enzyme histidine decarboxylase (HDC, EC 4.1.1.22) from *Lactobacillus 30a*, in the presence of cofactor PLP. This enzyme introduces deuterium or tritium from incubation medium (D_2_O or HTO) directly into the (*αR*) position of corresponding amine (Fig. 37).

Isotopomers of *N*^π^-methylhistamine, πMeHA (**42**) specifically labeled with hydrogen isotopes in the (*αR*) position i.e., [(*αR*)-^2^H]- (**42a**) and [(*αR*)-^3^H]-πMeHA (**42b**) were obtained in the same manner as in Fig. 37 by enzymatic decarboxylation of N^π^-methyl-l-histidine [138].

**Fig. 38:**
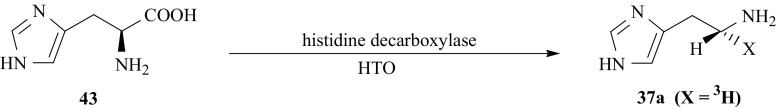
Synthesis of [(*αR*)-^3^H]-HA

Enzymatic decarboxylation of native l-histidine (**43**) carried out in incubation medium containing HTO leads to formation of [(*αR*)-^3^H]-HA (**37a**) [139] (Fig. 38). In the literature, there are also reports on the synthesis of [2-^2^H]-, and [2-^3^H]-HA and doubly labeled with tritium and ^14^C [2-^2^H, 2-^14^C]-HA obtained by decarboxylation of labeled l-His catalyzed by the enzyme HDC [46, 140].

The characteristic of HA (**37**) isotopomers are collected in Table 7.

**Table 7:** The characteristic of HA isotopomers

Compound	Specific activity (Bq/mmol)	Chemical yield (%)	References
[(*αR*)-^2^H]- τMeHA (**38a**)	–	58	[138]
[(*αR*)-^3^H]-τMeHA (**38b**)	2.8 × 10^6^	35	[138]
[(*αR*)-^2^H]- πMeHA (**42a**)	–	66	[138]
[(*αR*)-^3^H]-πMeHA (**42b**)	1.41 × 10^7^	81	[138]
[(*αR*)-^3^H]-HA (**37b**)	2.2 × 10^7^	88	[139]

### Synthesis of phenylpyruvic acid labeled with hydrogen and carbon isotopes

Phenylpyruvic acid, PPA (**3**) is a product of oxidative deamination reaction of l-Phe (**1**) presented in Fig. 2. In the course of this reaction some tautomerization of PPA takes place, and in the process the stereospecific abstraction of proton from 3-position of PPA is involved [141]. The numerical values of isotope effects allowed us to elucidate the intrinsic details of this mechanism. This kind of studies require the use of isotopomers of PPA labeled with deuterium and tritium in 3 position, and also the ^14^C-labeled isotopomer of PPA used as internal radiometric standard in the course of KIE assays. In the literature there are a few papers that describe the synthesis of deuterium-, [141] tritium-, [142] and ^14^C-labeled [143] isotopomers of PPA. Most of them yielding isotopomers bearing the label in position not useful for study of mechanism of reaction presented in Fig. 2 using KIE and SIE methods.

**Fig. 39:**
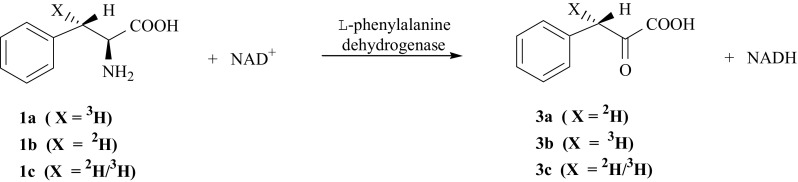
Synthesis of phenylpyruvic acid isotopomers labeled with hydrogen isotopes in (3*S*) position

Desired isotopomers of (**3**) labeled with isotopes of hydrogen were synthesized according to the reaction route shown in Fig. 39. Three isotopomers of l-Phe i.e., [(*3S*)-^2^H]- (**1b**), [(*3S*)-^3^H]-l-Phe (**1a**) [15] and [(*3S*)-^2^H/^3^H]-l-Phe (**1c**) [144] were converted into corresponding isotopomers of PPA, i.e., [(*3S*)-^2^H]- (**3a**), [(*3S*)-^3^H]- (**3b**), and [(*3S*)-^2^H/^3^H]-PPA (**3c**) by oxidative deamination, catalyzed by enzyme l-phenylalanine dehydrogenase (PheDH, EC 1.4.1.20) [144].

Isotopomer [1-^14^C]-PPA (**3d**) was synthesized as above using [1-^14^C]-l-Phe (**1** **h**) as a substrate [144].

The characteristic of PPA (**3**) isotopomers are collected in Table 8.

**Table 8:** The characteristic of PPA isotopomers

Compound	Specific activity (Bq/mmol)	Chemical yield (%)	References
[(*3S*)-^2^H]-PPA (**3a**)	–	48	[144]
[(*3S*)-^3^H]-PPA (**3b**)	7.32 × 10^7^	56	[144]
[(*3S*)-^2^H/^3^H]-PPA (**3c**)	3.7 × 10^6^	46	[144]
[1-^14^C]-PPA (**3d**)	7.1 × 10^6^	40	[144]

### Synthesis of halogen derivatives of l-Phe, l-Tyr and l-Trp labeled with hydrogen isotopes

Halogenated derivatives of l-Phe (**1**), l-Tyr (**14**) and l-Trp (**29**), labeled with short-lived radioisotopes i.e., ^18^F, ^123^I, ^125^I or ^76^Br are recently applied in nuclear medicine for diagnosis of many types of tumours and neurodegenerative diseases using positron emission tomography (PET) or single-photon emission computed tomography (SPECT). 2′-[^18^F]fluoro-l-Tyr is used for glioma imaging [145]. 2′-[^76^Br]bromo-α-methyl-l-Phe is a potential PET tumor tracer [146]. 3′-[^125^I]iodo-α-methyl-l-Tyr as well as 4′-[^123^I]iodo-l-Phe are validated for visualization by SPECT [147, 148] and 5′-[^18^F]fluoro-α-methyl-l-Trp holds great potential for cancer imaging using PET [149]. From a medical perspective, it is crucial to elucidate the influence of halogen substituents on kinetics of metabolic pathways of l-Phe (**1**), l-Tyr (**14**) and l-Trp (**29**) using KIE and SIE studies.

**Fig. 40:**
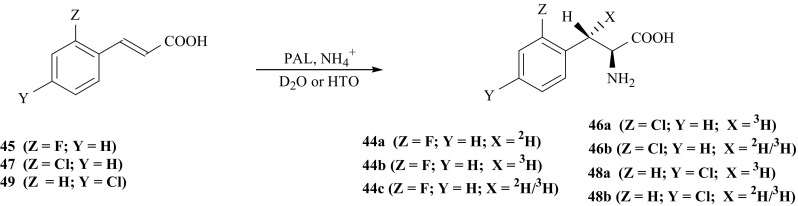
Enzymatic synthesis of halogen derivatives of l-Phe labeled with hydrogen izotopes

For KIE studies isotopomers of 2′-fluoro-l-Phe (**44**) i.e., 2′-fluoro-[(*3S*)-^2^H]- (**44a**), 2′-fluoro-[(*3S*)-^3^H]- (**44b**) and 2′-fluoro-[(*3S*)-^2^H/^3^H]-l-Phe (**44c**) were synthesized according to Fig. 40. Addition of ammonia to 2′-fluoro-(*E*)-cinnamic acid (**45**), catalyzed by PAL, was carried out in deuterated or tritiated buffer solutions [150, 151].

The same procedure was applied for synthesis of 2′-chloro-l-Phe (**46**) isotopomers, labeled with tritium and doubly labeled with deuterium and tritium in the (*3S*) position of the side chain i.e., 2′-chloro-[(*3S*)-^3^H]- (**46a**) and 2′-chloro-[(*3S*)-^2^H/^3^H]-l-Phe (**46b**). In case of this synthesis 2′-chloro-(*E*)-cinnamic acid (**47**) was used as a substrate. The isotopomers of 4′-chloro-l-Phe (**48**) were synthesized the same way using 4′-chloro-(*E*)-cinnamic acid (**49**) as substrate, yielding 4′-chloro-[(*3S*)-^3^H]- (**48a**) and 4′-chloro-[(*3S*)-^2^H/^3^H]-l-Phe (**48b**) [151] Fig. 40.

The characteristic of halogenated derivatives of l-Phe (**44**, **46**, **48**) are collected in Table 9.

**Table 9:** The characteristic of halogenated l-Phe isotopomers

Compound	Specific activity (Bq/mmol)	Chemical yield (%)	References
2′-fluoro-[(*3S*)-^2^H]-l-Phe (**44a**)	–	54	[150]
2′-fluoro-[(*3S*)-^3^H]-l-Phe (**44b**)	5.7 × 10^7^	40	[151]
2′-fluoro-[(*3S*)-^2^H/^3^H]-l-Phe (**44c**)	4.5 × 10^6^	39	[151]
2′-chloro-[(*3S*)-^3^H]-l-Phe (**46a**)	5.5 × 10^7^	45	[151]
2′-chloro-[(*3S*)-^2^H/^3^H]-l-Phe (**46b**)	1.4 × 10^7^	40	[151]
4′-chloro-[(*3S*)-^3^H]-l-Phe (**48a**)	7.1 × 10^7^	48	[151]
4′-chloro-[(*3S*)-^2^H/^3^H]-l-Phe (**48b**)	1.86 × 10^7^	38	[151]

**Fig. 41:**
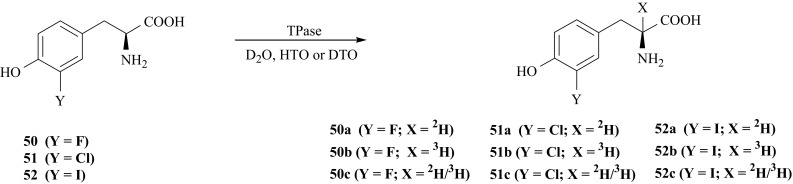
Enzymatic synthesis of halogen derivatives of l-Tyr labeled with hydrogen izotopes in the side chain

Desired isotopomers of 3′-fluoro- (**50**), 3′-chloro- (**51**) and 3′-iodo-l-Tyr (**52**) i.e., 3′-fluoro-[2-^2^H]- (**50a**), 3′-fluoro-[2-^3^H]- (**50b**), 3′-fluoro-[2-^2^H/^3^H]- (**50c**), 3′-chloro-[2-^2^H]- (**51a**), 3′-chloro-[2-^3^H]- (**51b**), 3′-chloro-[2-^2^H/^3^H]- (**51c**), 3′-iodo-[2-^2^H]- (**52a**), 3′-iodo-[2-^3^H]- (**52b**), 3′-iodo-[2-^2^H/^3^H]-l-Tyr (**52c**),were synthesized by isotopic exchange between (**50**), (**51**) or (**52**) and deuterated or tritiated water [150–152], catalyzed by enzyme TPase according to Fig. 41. This enzyme causes labilization of hydrogen in the second position of the side chain of various aromatic amino acids [53] and facilitates isotopic exchange.

**Fig. 42:**
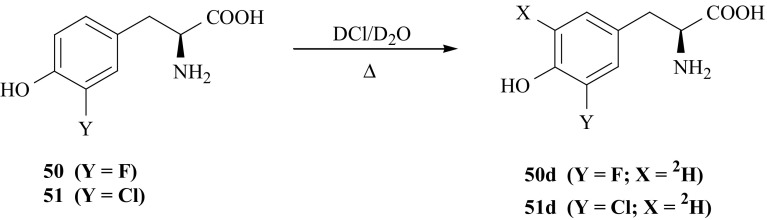
Synthesis of halogen derivatives of l-Tyr ring labeled with deuterium

Ring labelled isotopomers of (**50**) and (**51**) i.e., 3′-fluoro-[5′-^2^H]- (**50d**) and 3′-chloro-[5′-^2^H]-l-Tyr (**51d**) were synthesized by acid catalyzed isotopic exchange between (**50**) and (**51**) and deuterium from incubation medium at high temperature [153], Fig. 42.

The structure of the compounds was checked by ^1^H NMR spectroscopy. Obtained data revealed that deuterium incorporation takes place only at 5′ position of (**50**) and (**51**) aromatic ring and reached close to 100% deuterium enrichment.

The characteristic of halogenated derivatives of l-Tyr (**50**, **51**, **52**) are collected in Table 10.

**Table 10:** The characteristic of halogenated l-Tyr isotopomers

Compound	Specific activity (Bq/mmol)	Chemical yield (%)	References
3′-fluoro-[2-^2^H]-l-Tyr (**50a**)	–	98	[150]
3′-fluoro-[2-^3^H]-l-Tyr (**50b**)	6.8 × 10^6^	88	[151]
3′-fluoro-[2-^2^H/^3^H]-l-Tyr (**50c**)	5.7 × 10^6^	84	[151]
3′-fluoro-[5′-^2^H]-l-Tyr (**50d**)	–	58	[153]
3′-chloro-[2-^2^H]-l-Tyr (**51a**)	–	97	[150]
3′-chloro-[2-^3^H]-l-Tyr (**51b**)	6.1 × 10^6^	92	[151]
3′-chloro-[2-^2^H/^3^H]-l-Tyr (**51c**)	5.2 × 10^6^	86	[151]
3′-chloro-[5′-^2^H]-l-Tyr (**51d**)	–	70	[153]
3′-iodo-[2-^2^H]-l-Tyr (**52a**)	–	68	[152]
3′-iodo-[2-^3^H]-l-Tyr (**52b**)	5.73 × 10^6^	75	[152]
3′-iodo-[2-^2^H/^3^H]-l-Tyr (**52c**)	1.77 × 10^6^	97	[152]

**Fig. 43:**
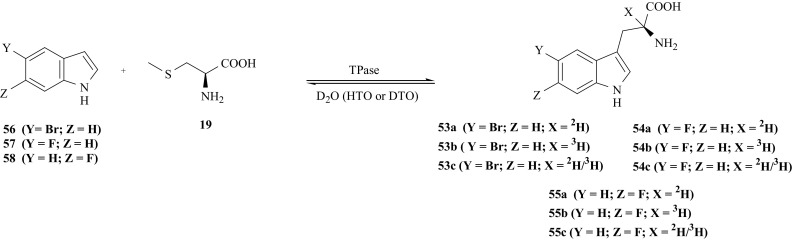
Enzymatic synthesis of halogen derivatives of l-Trp labeled with hydrogen izotopes

We have also developed the method for synthesis of halogenated derivatives of l-Trp (**29**), selectively labeled with hydrogen isotopes at the α-position of the side chain i.e., 5′-bromo-[2-^2^H]- (**53a**), 5′-bromo-[2-^3^H]- (**53b**), 5′-bromo-[2-^2^H/^3^H]- (**53c**), 5′-fluoro-[2-^2^H]- (**54a**), 5′-fluoro-[2-^3^H]- (**54b**), 5′-fluoro-[2-^2^H/^3^H]- (**54c**), 6′-fluoro-[2-^2^H]- (**55a**), 6′-fluoro-[2-^3^H]- (**55b**) and 6′-fluoro-[2-^2^H/^3^H]-l-Trp (**55c**). Coupling of the *S*-methyl-l-cysteine (**19**) with 5-bromo- (**56**), 5-fluoro- (**57**) or 6-fluoroindole (**58**) was catalyzed by TPase [150, 151] and carried out in deuterated or tritiated incubation medium, according to Fig. 43. In all cases 2-mercaptoethanol was used to prevent the growth of bacteria and fungi during incubation.

The characteristic of halogenated derivatives of l-Trp (**53**, **54**, **55**) are collected in Table 11.

**Table 11:** The characteristic of halogenated l-Trp isotopomers

Compound	Specific activity (Bq/mmol)	Chemical yield (%)	References
5′-bromo-[2-^2^H]-l-Trp (**53a**)	–	27	[150]
5′-bromo-[2-^3^H]-l-Trp (**53b**)	3.5 × 10^8^	35	[151]
5′-bromo-[2-^2^H/^3^H]-l-Trp (**53c**)	6.1 × 10^7^	22	[151]
5′-fluoro-[2-^2^H]-l-Trp (**54a**)	–	23	[150]
5′-fluoro-[2-^3^H]-l-Trp (**54b**)	3 × 10^8^	38	[151]
5′-fluoro-[2-^2^H/^3^H]-l-Trp (**54c**)	3 × 10^7^	32	[151]
6′-fluoro-[2-^2^H]-l-Trp (**55a**)	–	23	[150]
6′-fluoro-[2-^3^H]-l-Trp (**55b**)	3.2 × 10^8^	34	[151]
6′-fluoro-[2-^2^H/^3^H]-l-Trp (**55c**)	2.1 × 10^7^	29	[151]

## Conclusions

Taking into account the advantages of enzymatic synthetic methods, it is foreseeable that this type of reactions will gain a stronger presence in preparation of biologically active labeled compounds. While introduction of isotopic carbon atom to the backbone of a molecule may create some synthesis challenges, in majority of cases however, enzymatic syntheses are still easier to carry out than classic multistep organic syntheses. Furthermore this issue is greatly minimized when dealing with the substitution of the stable atom for radioactive one bonded to backbone of molecule (either isotopes of hydrogen and halogens) or addition of functional group bearing isotopic (for instance ^11^C- ^13^C- or ^14^C-) label.

## Abbreviations

Ac: Acetyl group (CH_3_CO–)
D: Deuterium, ^2^H
DA: Dopamine
d-AAO: d-amino acid oxidase
DAO: Diamine oxidase
EC: Enzyme Commission
FAD: Flavine adenine dinucleotyde
GPT: Glutamic-pyruvate transaminase
HA: Histamine
HDC: Histidine decarboxylase
KIE: Kinetic isotope effect
l-DOPA: 3′,4′-Dihydroxy-l-phenylalanine
l-His: l-Histidine
l-Phe: l-Phenylalanine
l-Trp: l-Tryptophan
5′-OH-l-Trp: 5′-Hydroxy-l-tryptophan
l-Tyr: l-Tyrosine
NAD^+^, NADH: Nicotinamide adenine dinucleotide (oxidized or reduced form)
PAL: Phenylalanine ammonia lyase
PET: Positron emission tomography
Ph: Phenyl
PheDH: Phenylalanine dehydrogenase
PKU: Phenylketonuria
PLP: Pyridoxal 5′-phosphate
PPA: Phenylpyruvic acid
SIE: Solvent isotope effect
SPECT: Single-photon emission computed tomography
T: Tritium, ^3^H
TA: Tyramine
TPase: Tryptophanase
YADH: Yeast alcohol dehydrogenase
πMeHA: *N*^π^-Methylhistamine
τMeHA: *N*^τ^-Methylhistamine
